# Real World First-Line Treatments and Outcomes of Nab-Paclitaxel Plus Gemcitabine, mFOLFIRINOX and GEMOX in Unresectable Pancreatic Cancer from a Chinese Single Institution

**DOI:** 10.3390/curroncol28010023

**Published:** 2020-12-30

**Authors:** Qi Quan, Yixing Wang, Fenghua Wang, Dongsheng Zhang, Xiuxing Chen, Wenzhuo He, Bei Zhang, Guifang Guo

**Affiliations:** 1 State Key Laboratory of Oncology in South China, Collaborative Innovation Center for Cancer Medicine, Guangzhou 510060, China; quanqi@sysucc.org.cn (Q.Q.); sysuyixing@163.com (Y.W.); wangfh@sysucc.org.cn (F.W.); zhangdsh@sysucc.org.cn (D.Z.); chenxiux@sysucc.org.cn (X.C.); hewzh@sysucc.org.cn (W.H.); 2 VIP Region, Sun Yat-Sen University Cancer Center, Guangzhou 510060, China; 3 Department of Medical Oncology, Sun Yat-Sen University Cancer Center, Guangzhou 510060, China

**Keywords:** pancreatic cancer, chemotherapy, nab-paclitaxel plus gemcitabine, mFOLFIRINOX, GEMOX

## Abstract

Background: There have not been any head-to-head prospective studies to compare the effects of different chemotherapy regimens as first-line treatments for unresectable pancreatic cancer (UPC). We aimed to compare the effectiveness of nab-paclitaxel plus gemcitabine, mFOLFIRINOX and gemcitabine plus oxaliplatin (GEMOX) as first-line treatments by using real-world data from Chinese patients. Methods: We retrospectively included patients with UPC treated with nab-paclitaxel plus gemcitabine, mFOLFIRINOX or GEMOX as a first-line treatment at Sun Yat-sen University Cancer Center. Overall survival (OS), progression-free survival (PFS), objective response rate (ORR) and disease control rate (DCR) were assessed. Results: A total of 117 patients were administered nab-paclitaxel plus gemcitabine (*n* = 62), mFOLFIRINOX (*n* = 30) or GEMOX (*n* = 25) as first-line chemotherapy. The median OS was 11.1, 10.1 and 10.2 months (*p* = 0.75) in the nab-paclitaxel plus gemcitabine, mFOLFIRINOX and GEMOX, respectively. The ORR was similar among the three groups (24%, 23% and 32%, *p* = 0.76) and the DCR was higher in the nab-paclitaxel-gemcitabine group (82%) than the other two groups (60% and 64%, *p* = 0.04). The most common adverse events of grade 3 or 4 were neutropenia (32%, 28% and 5%), peripheral neuropathy (13%, 16% and 0) and fatigue (9%, 16% and 5%). Febrile neutropenia occurred in 2%, 4% and 5% of the patients in the three groups. Conclusion: In the first line treatment of UPC, our results suggest that nab-paclitaxel plus gemcitabine was associated with a higher DCR than mFOLFIRINOX or GEMOX, while all groups demonstrated similar OS, PFS and ORR.

## 1. Introduction

Pancreatic adenocarcinoma is the sixth leading cause of cancer death in China, with 116,291 new patients and 110,390 deaths in 2018. It is the seventh most common cause of death from cancer worldwide [1].

In recent decades, fluorouracil was the first acknowledged systemic chemotherapy for unresectable pancreatic cancer (UPC). Gemcitabine replaced fluorouracil as first-line chemotherapy for patients with UPC in 1997 according to a phase III study with a result of longer OS (5.6 vs 4.4 months, *p* = 0.002) [2]. Gemcitabine combined with oxaliplatin (GEMOX) was the first combined treatment shown to be superior to gemcitabine alone from the clinical benefit, objective response rate (ORR) and progression-free survival (PFS) perspective, although it failed to give evidence of a statistically significant advantage in terms of overall survival (OS, 9.0 versus 7.1 months; *p* = 0.13) [3]. In 2011, a regimen containing oxaliplatin, irinotecan, fluorouracil, and leucovorin (FOLFIRINOX) was introduced as a new first-line regimen for patients with UPC that extended survival time by 4.3 months compared to treatment with gemcitabine alone [4]. FOLFIRINOX treatment is mainly used for patients with better physical status due to more toxicities and frequently required growth factor treatment. A modified version of FOLFIRINOX (mFOLFIRINOX) is widely used in real world settings that utilizes reduced doses of some components and has an improved safety profile with similar efficacy [5,6]. In the MPACT trial published in 2013, a combination treatment of nab-paclitaxel and gemcitabine achieved better OS and ORR compared to gemcitabine alone (OS, 8.7 versus 6.6 months; *p* < 0.001), resulting in the regulatory approval of nab-paclitaxel plus gemcitabine as an optional measure for patients with UPC [7,8]. Nab-paclitaxel combined with gemcitabine, because it is better tolerated, is often used by Chinese physicians, although nab-paclitaxel has higher drug acquisition costs.

Which combined chemotherapy is the preferred option for first-line treatment? Some scholars in other countries or regions have compared the efficacies of nab-paclitaxel plus gemcitabine and FOLFIRINOX in the clinical setting retrospectively [9,10,11]. A study in Europe found that a combination of chemotherapeutics improved survival with no significant differences observed between nab-paclitaxel in combination with gemcitabine and FOLFIRINOX [9]. Kang et al. from Korea reported that the nab-paclitaxel in combination with gemcitabine and FOLFIRINOX exhibited equally matched ORR (34% vs 34%, *p* = 0.88) and median PFS (6.8 vs 5.1 months, *p* = 0.19) in a retrospective study, in which nab-paclitaxel plus gemcitabine had a better OS (11.4 vs 9.6 months, *p* = 0.002) [10]. The comparison of GEMOX and other combination therapies has not been previously reported.

Approved treatment options for UPC are limited, and the random clinical trial to directly compare the effect and adverse events of standard regimens is no available, so the analysis of real-world treatment outcomes can provide valuable information for making treatment decisions. We reviewed the first-line options for patients who have undergone palliative chemotherapy in the past ten years in our hospital and compared the effectiveness of nab-paclitaxel in combination with gemcitabine, mFOLFIRINOX and GEMOX, which are three commonly used chemotherapy options for patients with good performance status in a real-world setting.

## 2. Patients and Methods

### 2.1. Patient Inclusion

The study complied with the standards of the Declaration of Helsinki and was approved by the Research Ethics Committee of Sun Yat-Sen University Cancer Center (number: B2018-060-01; date: 13 August 2018).

Patients pathologically diagnosed with pancreatic cancer were enrolled between March 2005 and March 2018 at our treatment center. The main inclusion criteria include: (1) patients had a cytological or histological diagnosis of pancreatic ductal adenocarcinoma and CT or MRI to confirm the unresectable disease, (2) patients received one of three regimens, nab-paclitaxel plus gemcitabine, mFOLFIRINOX or GEMOX, as first-line treatment, and (3) patients achieved no less than two rounds of treatment.

Patients if they received less than two cycles of first-line treatment or did not have imaging evaluation records were not suitable for this study.

### 2.2. Patient Information Collection

The following clinical information was collected and recorded: age, sex, tumor location, stage, node stage, tumor histological grade, serum carcinoembryonic antigen (CEA), serum CA19-9, frequency of first-line chemotherapy, and the efficacy and toxicity of chemotherapy.

### 2.3. Treatment and Response Assessment

GEMOX treatment consisted of an intravenous infusion of gemcitabine (1000 mg/m^2^) on days 1, 8 and an infusion of oxaliplatin (85 mg/m^2^) once every three weeks. mFOLFIRINOX treatment consisted of a 3-h intravenous infusion of oxaliplatin (65 mg/m^2^) followed by a 90-min intravenous infusion of irinotecan (150 mg/m^2^) and a 46-h uninterrupted infusion of fluorouracil (2400 mg/m^2^) that was carried out every two weeks, as recorded in previous clinical study [6]. Nab-paclitaxel in combination with gemcitabine treatment consisted of an intravenous infusion of nab-paclitaxel (125 mg/m^2^) followed by an infusion of gemcitabine (1000 mg/m^2^) on days 1, 8 and every three weeks thereafter. The assessment for tumor response was performed after 2 cycles for both Nab-paclitaxel plus gemcitabine and GEMOX and after 4 cycles for mFOLFIRINOX by radiologists following Response Evaluation Criteria in Solid Tumors (RECIST) version 1.1.

### 2.4. Statistical Analysis

OS was the interval time between the first day of chemotherapy and the day of death, whereas PFS was time separation from the first day of chemotherapy to disease progression or death from any cause; the ORR was defined as the percentage of patients with a confirmed complete or partial response; the DCR is defined as the percentage of complete response, partial response and stability of total patients. We performed statistical analysis using SPSS version 22.0 (IBM Corporation, Armonk, NY, USA). The chi-square test was used for categorical variable statistics. The Kaplan-Meier and the log-rank test were adopted for survival analyses. A two-tailed *p*-value < 0.05 was recognized statistically significant.

## 3. Results

### 3.1. Patient Characteristics

A total of 117 patients who were accessible for survival analysis were enrolled in this study from March 2005 to March 2018, including 62 patients in the nab-paclitaxel-gemcitabine group, 30 patients in the mFOLFIRINOX group, and 25 patients in the GEMOX group.

The median age of the included patients was 55 years (57 years in the nab-paclitaxel plus gemcitabine and mFOLFIRINOX, 51 years in the GEMOX), of which 72% were male. Performance status (PS) is satisfactory (0 or 1) for most patients (*n* = 104, 88.9%). Forty-four patients (37.6%) had lesions in the head of the pancreas, and the remaining patients were in the body or tail of the pancreas (*n* = 73, 62%). The liver was the most frequently occurring metastatic site (*n* = 80, 68.4%). The number of patients with liver metastases in groups of nab-paclitaxel plus gemcitabine, mFOLFIRINOX and GEMOX were 32 (52%), 29 (97%) and 19 (76%). The median value of serum CA19-9 of the included patients was 629.5 U/mL (range = 1–20,000). One hundred and four patients presented with metastatic disease. The clinical features of patients in the three groups were balanced (Table 1), although there were more stage III patients in the nab-paclitaxel-gemcitabine group than in the other two groups (*p* = 0.05).

### 3.2. Efficacy and Survival

Table 2 provides a summary of the treatments administered in each study arm. The final follow-up time was 1 March 2019. The median number of chemotherapy rounds administered was 4 (range, 2 to 12) in the nab-paclitaxel-gemcitabine group, 6 (range, 2 to12) in the mFOLFIRINOX group, and 4 (range, 2 to 8) in the GEMOX group. The median duration of treatment was 4.0 months in the nab-paclitaxel–gemcitabine group, 3.0 months in the mFOLFIRINOX group, and 2.9 months in the GEMOX group, with 32%, 20% and 24% of patients, respectively, receiving treatment for at least 6 months.

In the nab-paclitaxel-gemcitabine group, 25% of the patients had reductions in the nab-paclitaxel dose and 28% had reductions in the gemcitabine dose. In the mFOLFIRINOX group, 16% of the patients had reductions in the irinotecan, oxaliplatin and fluorouracil dose. In the GEMOX group, 18% of the patients had reductions in the gemcitabine dose and 5% had reductions in the oxaliplatin dose.

The rate of objective response in the patients was comparable in the three treatment groups; it was 24% (15 of 62 patients) in the nab-paclitaxel-gemcitabine group, 27% (8 of 30 patients) in the mFOLFIRINOX group, and 32% (8 of 25 patients) in the GEMOX group. Patients administered with nab-paclitaxel in combination with gemcitabine achieved a higher likelihood of stable disease than the other two groups; the likelihood was 58% for nab-paclitaxel in combination with gemcitabine versus 33% for mFOLFIRINOX and 32% for the GEMOX (*p* = 0.02). The group of nab-paclitaxel in combination with gemcitabine had a significantly higher disease control rate (DCR) than the other two groups; it was 82% versus 60% and 64% in the mFOLFIRINOX and GEMOX groups, respectively (*p* = 0.04). The DCR of the two groups treated with mFOLFIRINOX and GEMOX was comparable (*p* = 0.79).

**Figure 1:**
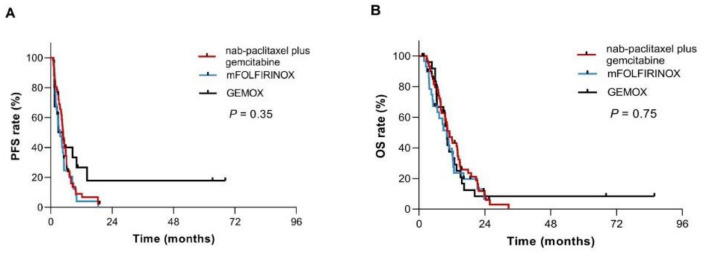
Progression-free survival (PFS) and overall survival (OS) for patients with unresectable pancreatic cancer according to first-line chemotherapy regimens. (**A**) Kaplan-Meier plot of PFS according to the first-line chemotherapy regimens with nab-paclitaxel plus gemcitabine, mFOLFIRINOX and GEMOX; (**B**) Kaplan-Meier plots of OS according to first-line chemotherapy regimens with nab-paclitaxel plus gemcitabine, mFOLFIRINOX and GEMOX.

Until the final follow-up time, one hundred and twelve patients showed tumor progression after first-line chemotherapy, including fifty-nine, twenty-nine, twenty-four patients in the nab-paclitaxel combined with gemcitabine, mFOLFIRINOX and GEMOX groups, respectively. Increased PFS was observed in the nab-paclitaxel combined with gemcitabine group compared to that in the other two groups, with a median value of 4.9 months (95% CI 4.1–5.6) observed in the nab-paclitaxel-gemcitabine group versus 3.7 months (95% CI, 3.6 to 4.0) in the FOLFIRINOX group and 4.7 months (95% CI 2.0–7.4) in the GEMOX group (*p* = 0.35, Figure 1A). As of the follow-up date, ninety-four patients had died, including forty-nine, twenty-three and twenty-two patients in the groups treated with nab-paclitaxel plus gemcitabine, mFOLFIRINOX and GEMOX, respectively. The median OS was superior in the nab-paclitaxel plus gemcitabine cohort than in the mFOLFIRINOX and GEMOX cohorts; it was 11.1 months (95% CI 8.9–13.4) versus 10.1 months (95% CI 6.5–13.6) and 10.2 months (95% CI 9.2–11.2), respectively (*p* = 0.75, Figure 1B). No statistically significant *p* values were found between any two treatment groups for PFS and OS. The 6-month, 1-year and 2-year survival rates were also higher in the nab-paclitaxel-gemcitabine group than in the other two groups. The survival rates were 47%, 39.6% and 37.6% in the nab-paclitaxel-gemcitabine group, the mFOLFIRINOX group and the GEMOX group, respectively, at 1 year.

### 3.3. Subsequent Anticancer Treament

Data on subsequent second-line chemotherapy after failure (disease progression or intolerability) of first-line were available for all patients. The proportion of the use of second-line therapy was balanced among the three programs: 55% in the nab-paclitaxel–gemcitabine group, 37% in the mFOLFIRINOX group and 48% in the GEMOX group (*p* = 0.26). Thirty-four patients who received second-line treatment in the AG regimen were as follows: mFOLFIRINOX or S-1, oxaliplatin, and irinotecan (SOXIRI) were given in 44.1% (15/34) of patients. 5-FU monotherapy or in combination with oxaliplatin were given in 35.3% (12/34) of patients, and immune checkpoint inhibitors were given in in combination with chemotherapy in 18.2% (3/55) of patients. Fluorouracil-based regimens were given in 79.4% (27/34) of patients in the AG group and in 75.0% (9 /12) of patients in the GEMOX group. Gemcitabine-based regimens were given in 72.7% (8/11) of patients in the mFOLFIRINOX group. Four patients received local radiotherapy in the 13 locally advanced patients.

### 3.4. Univariate and Multivariate Analyses

**Figure 2:**
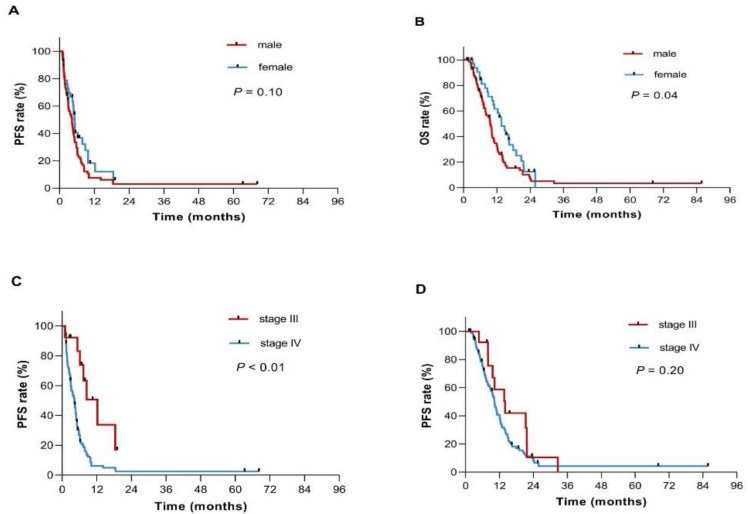
Progression-free survival (PFS) and Overall survival (OS) for patients with unresectable pancreatic cancer in the subgroups. (**A**,**B**) Kaplan-Meier plots of PFS and OS according to gender; (**C**,**D**) Kaplan-Meier plots of PFS and OS according to clinical stage.

Factors with significant prognostic significance in univariate analysis were included in multivariate analysis (*p* < 0.20). As shown in Table 3, male, liver metastasis, metastatic lesions ≥2 and stage significantly correlated with reduced OS and PFS by univariate analysis (Figure 2). Age ≥65 years and CA19-9 level were potentially associated with poor OS but not significantly associated with PFS. In the multivariate analyses, gender was an independent prognostic indicator of OS (relative risk: 1.79, 95% CI: 1.03–2.83, *p* = 0.04) and PFS (relative risk: 1.82, 95% CI: 1.09–3.02, *p* = 0.02). Male patients had a significantly higher risk of progression and death than female patients. In addition, stage was also considered an independent prognostic factor of PFS (relative risk: 2.61, 95% CI: 1.05–6.49, *p* = 0.04).

### 3.5. Safety

Treatment-emergent adverse events were recorded in 103 patients, including 56 patients with the nab-paclitaxel combined with gemcitabine group, 25 patients with mFOLFIRINOX group, and 22 patients with GEMOX group. Grade 3 or 4 adverse events with high frequency were neutropenia (32% in the nab-paclitaxel in combination with gemcitabine group, 28% in the mFOLFIRINOX group and 5% in the GEMOX group), peripheral neuropathy (13%, 16% and 0%, respectively) and fatigue (21%, 16% and 16%, respectively), as shown in Table 4. No sepsis occurred in the three groups of patients. The incidence rate of adverse events of grade 3 or higher among the three groups was similar (*p* = 0.50).

## 4. Discussion

The present retrospective analysis showed that nab-paclitaxel plus gemcitabine, mFOLFIRINOX and GEMOX may have had comparable efficacy in terms of outcomes for patients with UPC in a clinical setting at our therapeutic center.

Researchers at our center reported that the nab-paclitaxel plus gemcitabine regimen resulted in the enhanced survival of Chinese patients with metastatic pancreatic cancer in a previous I/II trial [12,13]. The National Comprehensive Cancer Network guidelines put forward that the combination of nab-paclitaxel and gemcitabine as the preferred chemotherapy regimen of patients with metastatic pancreatic cancer [14]. In our research, nab-paclitaxel plus gemcitabine showed a higher DCR than the other two groups. A potential reason for this is that a higher proportion of patients in the nab-paclitaxel-gemcitabine group had stage III disease than in the mFOLFIRINOX and GEMOX groups, which has been found correlated to improved survival. We have performed a subgroup analysis on patients with different stages considering that the prognosis of pancreatic adenocarcinoma is highly influenced by the stage at diagnosis. The results suggested that there was no significant difference in PFS and OS among the three groups of patients with IV stage. The survival benefits of the nab-paclitaxel plus gemcitabine in our practice study appeared to be better than those observed in the MPACT trial (median OS: 11.1 vs 8.5 months). The possible reason is that patients in our study have a higher proportion of favorable features, including 27% of patients with age >75 years versus 41% in the MPACT trial and 52% of patients who had liver metastasis versus 85% in the MPACT trial. On the other hand, patients administered with nab-paclitaxel plus gemcitabine appeared to have higher economic status and received better supportive care according to clinical observation. Nab-paclitaxel plus gemcitabine produced a greater than 80% of DCR, as was found in previous studies conducted during a stage II clinical trial at our hospital and at a clinical practice in Korea [10,13], in which the DCR was 80.9% and 82.0%, respectively. The practice effects with nab-paclitaxel plus gemcitabine were consistent with previous retrospective analysis by others [15,16].

In our analysis, the mFOLFIRINOX showed inferior survival benefits compared to those reported in the PRODIGE4-ACCORD 11 study. The results might be elucidated by the less-than-optimal patient features of our study, considering that 87.6% of patients had liver metastasis and 62% were male in the PRODIGE 4/ACCORD 11 trial, while 96.7% of the patients in our study had liver metastasis and 73% were male. Both factors were associated with poor prognosis in our study. In addition, it has been argued that the study population of the ACCORD 11 trial was strictly selected [17]. Consistent with our results, previous retrospective studies showed reduced survival compared to that observed in the PRODIGE 4/ACCORD 11 trial. mFOLFIRINOX but not FOLFIRINOX was used for the patients with a PS of 0–1 in our clinical practice, which might also be partly responsible for the reduced efficacy. Patients treated with FOLFIRINOX need to be monitored for adverse effects more frequently and must be treated according to a strict schedule; otherwise, it is difficult to start the next cycle on time. However, given retrospective design, there is a high risk of selection bias, for mFOLFIRINOX group where there is a higher proportion of liver metastastases and stage IV patients than that in other two groups. Moreover, patients included in mFOLFIRINOX had less access to second line treatment. These factors may have affected survival outcome.

GEMOX treatment produced satisfactory survival as a first-line therapy and was well tolerated in patients according to our clinical experience. In line with expectations, GEMOX treatment was not inferior to the other two treatments in terms of PFS, which was with 4.7 months, and OS, which was 10.2 months, compared to the PFS of 5.8 months and the OS of 9.0 months observed in the GISCAD Trial. With respect to the rate of objective response, GEMOX showed even greater advantages over the other two treatments, as it resulted in an ORR of 32%. Therefore, GEMOX treatment is also a potential option for UPC. GEMOX showed amazing efficacy in a patient in our study by resulting in a disease-free survival of more than thirteen years; this patient is still alive currently and works two jobs. He presented with pancreatic cancer, portal vein tumor thrombus and liver metastasis in March 2006. Fortunately, the mass in the liver disappeared, and the other lesions decreased after six cycles of GEMOX as a first-line treatment. Subsequently, the patient achieved a complete response after gamma-knife radiotherapy with 45.5 Gy/13F administered to a local mass. Finally, 8 cycles of capecitabine were given as a maintenance treatment. Three studies [18,19] have published significantly improved survival and responses to platinum-based chemotherapy for BRCA-positive pancreatic cancers; therefore, gemcitabine plus cisplatin is recommended by the NCCN for UPC with BRCA1/2 or PABL2 mutation. Oxaliplatin or carboplatin is often used as a substitute for cisplatin in our clinical practice, according to reports from Johns Hopkins University (JHU)-affiliated hospitals and the M.D. Anderson Cancer Center [20]. It is regretful that our study did not determine the mutational status of BRCA1/2 or PABL2 for patients treated with GEMOX. The improved effects of GEMOX in the present study might be due to the inclusion of patients with higher rates of BRCA1/2 or PABL2 mutation; this topic needs further study.

In this study, male gender showed independent poor prognostic value for both PFS and OS. Survival in male patients is reduced compared to that in female patients, which might be explained by a higher percentage of male patients who had liver metastasis than female patients (73% vs 57%). Increased CA19-9 levels tended to be negatively correlated with survival. However, CA19-9 failed to show independent prognostic significance in the multivariate analyses, which was inconsistent with the results of previous studies [21,22,23], in which serum CA19-9 has been shown to have independent prognostic value. Since three-quarters of patients have an increased CA19-9 that is greater than the upper limit of the normal range, we chose the median as the cutoff value.

The safety profiles for the three regimens were consistent with those in previous research [3,4,7]. A higher percentage of patients in the nab-paclitaxel-gemcitabine group than in other groups had neutropenia of grade ≥ 3. The frequency of thrombocytopenia of grade ≥ 3 in the GEMOX group was higher than that in the other two groups. Unlike that observed in other studies, a lower percentage of patients in the mFOLFIRINOX group than the nab-paclitaxel plus gemcitabine or GEMOX groups had adverse events > grade 3 with hematological symptoms and fatigue. Fewer adverse events were due to reduced doses of chemotherapy drugs in the mFOLFIRINOX group, and some of the patients who used mFOLFIRINOX were administered granulocyte colony-stimulating factor (GCSF) in advance to prevent leukopenia. A limitation of the study was that a considerable number of patients had no complete record of adverse events, and quality of life was not evaluated.

The patient characteristics were similar in three groups, and all treatment options showed comparable efficacy in terms of outcomes in patients with UPC. Despite the improved ORR achieved with nab-paclitaxel plus gemcitabine, a considerable number of patients could not afford it because nab-paclitaxel is not yet included in Medicare in China and has a higher cost. In addition, the rates of thrombocytopenia were higher in the nab-paclitaxel-gemcitabine group than in the others. mFOLFIRINOX is mainly recommended for patients with good PS (0-1), and the rates of peripheral neuropathy were higher in the mFOLFIRINOX group than in the GEMOX group. Although GEMOX treatment is limited to pancreatic cancers with BRCA1/2 or PABL2 mutations according to the NCCN, it resulted in similar PFS and OS as the other two regimens in unselected Chinese patients.

Our research conclusion was limited with the inevitable bias brought by retrospective research and a small number of cases and to be verified with a larger sample and multi-center data in a prospective study.

## 5. Conclusions

Our study compared the efficacy, survival and safety profiles of commonly used front line chemo of nab-paclitaxel plus gemcitabine, mFOLFIRINOX and GEMOX of patients with unresectable pancreatic cancer in the real-world from a Chinese single institution. The results found DCR of nab-paclitaxel plus gemcitabine was higher than that of mFOLFIRINOX or GEMOX in the first-line treatment of UPC. There was no significant difference for OS, PFS and ORR of the three treatment groups.

## Figures and Tables

**Table 1:** Baseline characteristics among eligible patients with unresectable pancreatic cancer.

Characteristics	Nab-Paclitaxel-Gemcitabine	mFOLFIRINOX	GEMOX	*p*-Value
No. of cases	62	30	25	
Age at diagnosis (years)				0.23
<65	45(73)	26(87)	21(84)	
≥65	17(27)	4(13)	4(16)	
Gender				0.80
Male	43(69)	22(73)	19(76)	
Female	19(21)	8(27)	6(24)	
ECOG PS				0.28
0–1	53(85)	29(97)	22(88)	
2	9(15)	1(3)	3(12)	
Pancreatic tumor location				0.17
Head	19(31)	12(40)	13(52)	
Body/Tail	43(69)	18(60)	12(48)	
Liver metastasis				< 0.01
Yes	32(52)	29(97)	19(76)	
No	30(48)	1(3)	6(24)	
Number of metastatic sites				0.98
<2	42(68)	21(70)	17(68)	
≥2	20(32)	9(30)	8(32)	
diabetes				0.80
Yes	48(77)	25(83)	20(80)	
No	14(23)	5(17)	5(20)	
Tumor histological grade ^a^				0.14
High or moderate	20(44)	12(41)	4(19)	
Low	26(56)	17(59)	17(81)	
CA19–9 (ng/mL) ^b^				0.73
≤35	13(22)	5(17)	4(18)	
<35	45(78)	24(83)	18(82)	
Disease status on study (stage)				0.05
Locally advanced (III)	11(18)	1(3)	1(4)	
Metastatic (IV)	51(82)	29(97)	24(96)	
Second-line therapy				0.26
Yes	34(55)	11(37)	12(48)	
No	28(45)	19(63)	13(52)	

^a^ tumor histological grade recorded in 96 patients. ^b^ CA199 recorded in 109 patients. Abbreviations: PS, performance status; CEA, carcino-embryonic antigen; CA19-9, ca antigen 199.

**Table 2:** Efficacy of three different first-line regimens in patients with unresectable pancreatic cancer.

	Nab-Paclitaxel-Gemcitabine	mFOLFIRINOX	GEMOX	*p*-Value
Total cases	62	30	25	
treatment cycles (median)	4	6	4	
PR, *n* (%)	15(24)	8(27)	8(32)	0.76
SD, *n* (%)	36(58)	10(33)	8(32)	0.02
PD, *n* (%)	11(18)	12(40)	9(36)	0.04
ORR ^a^, *n* (%)	15(24)	8(27)	8(32)	0.76
DCR ^b^, *n* (%)	51(82)	18(60)	16(64)	0.04
Median PFS (months)	4.9	3.7	4.7	0.35
6 mo (%)	34.5	25.6	40.1	0.47
12 mo (%)	9.2	4.3	26.8	0.44
Median OS (months)	11.1	10.1	10.2	0.75
6 mo (%)	79.6	67.2	83.5	0.14
12 mo (%)	47.0	39.6	37.6	0.88
18 mo (%)	23.6	19.8	12.5	0.87
24 mo (%)	8.8	6.6	8.3	0.73

^a^ ORR is defined as the percentage of patients who had a CR or PR. ^b^ DCR is defined as the. percentage of patients who had a CR, PR, or SD. Abbreviations: PR, Partial response; SD, Stable disease; PD, Progressive disease; ORR, Rate of objective response; DCR, Rate of disease control; OS, overall survival; PFS, progression-free survival. Gem, gemcitabine.

**Table 3:** Results of univariate and multivariate analyses of prognostic factors for first-line PFS and OS in all eligible patients with unresectable pancreatic cancer.

Parameter	PFS			OS		
	Univariate Analysis	Multivariate Analysis		Univariate Analysis	Multivariate Analysis	
	*p*-value	RR (95% CI)	*p*-value	*p*-value	RR (95% CI)	*p*-value
Age ≥ 65 years	0.44			0.10		
Gender(male)	0.11	1.82(1.09–3.02)	0.02	0.04	1.79 (1.03–2.83)	0.04
ECOG PS ≥ 2	0.28			0.55		
Pancreatic tumor location	0.54			0.40		
Liver metastasis	0.01			0.06		
Number of metastatic sites ≥ 2	0.15			0.13		
diabetes CA19-9(>629.5 ng/mL)	0.21 0.80			0.96 0.05		
Stage IV	<0.01	2.61(1.05–6.49)	0.04	0.20		
Second-line chemotherapy	0.86			0.07		

Abbreviations: PFS progression-free survival, OS overall survival, RR relative risk, CI confidence interval, ECOG PS Eastern Cooperative Oncology Group performance status.

**Table 4:** Grade ≥ 3 treatment-emergent adverse events of three subgroups with different regimens inpatients with unresectable pancreatic cancer.

	Nab-Paclitaxel Plus Gem	mFOLFIRINOX	GEMOX
Patients assessed (*n*)	56	25	22
Total grade ≥ 3 AEs (*n*)	28	13	6
Hematologic AEs			
Neutropenia (%)	18(32)	7(28)	1(5)
Leukopenia (%)	14(25)	5(20)	1(5)
Thrombocytopenia (%)	2(4)	1(4)	4(18)
Anemia (%)	8(14)	7(28)	2(9)
Receipt of growth factors	12(21)	5(20)	2(9)
Febrile neutropenia	1(2)	1(4)	1(5)
Nonhematologic AEs ^a^			
Peripheral neuropathy (%)	7(13)	4(16)	0(0)
Fatigue (%)	5(9)	4(16)	1(5)

^a^ Nonhematologic adverse events with incidence greater than 5%. Abbreviations: AEs, adverse events.
